# Activation of the glucocorticoid receptor rapidly triggers calcium‐dependent serotonin release in vitro

**DOI:** 10.1111/cns.13634

**Published:** 2021-03-14

**Authors:** Nicolas Paul, Justine Raymond, Sara Lumbreras, Dusan Bartsch, Tillmann Weber, Thorsten Lau

**Affiliations:** ^1^ Department of Psychiatry and Psychotherapy Biochemical Laboratory Central Institute of Mental Health Medical Faculty Mannheim Heidelberg University Mannheim Germany; ^2^ Transgenic Models Central Institute of Mental Health Medical Faculty Mannheim Heidelberg University Mannheim Germany; ^3^ Department of Addictive Behavior and Addiction Medicine Central Institute of Mental Health Medical Faculty Mannheim Heidelberg University Mannheim Germany; ^4^ MEDIAN Klinik Wilhelmsheim Oppenweiler Germany; ^5^ Department of Translational Brain Research Central Institute of Mental Health Medical Faculty Mannheim Heidelberg University Mannheim Germany; ^6^ Hector Institute for Translational Brain Research Mannheim Germany; ^7^ German Cancer Research Center Heidelberg Germany; ^8^ Present address: Department of Anesthesiology and Operative Intensive Care Medicine (CCM, CVK Charité – Universitätsmedizin Berlin corporate member of Freie Universität Berlin Humboldt‐Universität zu Berlin and Berlin Institute of Health Augustenburger Platz 1 Berlin 13353 Germany

**Keywords:** calcium channels, glucocorticoid, neurotransmission, receptors, serotonergic neurons, serotonin

## Abstract

**Aims:**

Glucocorticoids rapidly provoke serotonin (5‐HT) release in vivo. We aimed to investigate molecular mechanisms of glucocorticoid receptor (GR)‐triggered 5‐HT release.

**Methods:**

Employing 1C11 cells to model 5‐HT neurotransmission, immunofluorescence and Pearson's Correlation Coefficient were used to analyze colocalization of GR, 5‐HT, vesicle membrane protein synaptotagmin 1 and vesicle dye FM4‐64FX. FFN511 and FM4‐64FX dyes as well as calcium imaging were used to visualize vesicular 5‐HT release upon application of GR agonist dexamethasone, GR antagonist mifepristone and voltage‐gated calcium channel (VGCC) inhibitors.

**Results:**

GR, 5‐HT, synaptotagmin 1 and FM4‐64FX showed overlapping staining patterns, with Pearson's Correlation Coefficient indicating colocalization. Similarly to potassium chloride, dexamethasone caused a release of FFN511 and uptake of FM4‐64FX, indicating vesicular 5‐HT release. Mifepristone, calcium depletion and inhibition of L‐type VGCC significantly diminished dexamethasone‐induced vesicular 5‐HT release.

**Conclusions:**

In close proximity to 5‐HT releasing sites, activated GR rapidly triggers L‐type VGCC‐dependent vesicular 5‐HT release. These findings provide a better understanding of the interrelationship between glucocorticoids and 5‐HT release.

## Graphical abstract

In proximity to serotonin‐containing vesicles, glucocorticoids (dexamethasone) bind to glucocorticoid receptors (1) and mediate opening of L‐type voltage‐gated calcium channels (VGCC; 2). Subsequent calcium influx (3) triggers rapid vesicular serotonin release (4). This mechanism is similar to depolarization‐induced serotonin release, which also depends on L‐type voltage‐gated calcium channels (5).

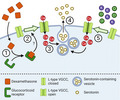

## 1 INTRODUCTION

The serotonin (5‐HT) system affects various physiological functions such as circadian rhythm, appetite, behavior and learning. Alterations of the 5‐HT system are believed to play a pivotal role in the pathogenesis of many psychiatric diseases such as depression.^1^

In serotonergic neurons, 5‐HT is synthesized from L‐tryptophan and packed into vesicles.^2^ Excitation of 5‐HT neurons triggers calcium influx via voltage‐gated calcium channels (VGCCs).^3^ At the center of the neurotransmitter release process, calcium ions then bind to the vesicle membrane‐bound calcium sensor synaptotagmin 1. Upon calcium binding, synaptotagmin 1 forms a complex with soluble NSF attachment protein receptors (SNAREs) and the plasma membrane, which causes vesicle fusion pore opening and subsequent neurotransmitter liberation.^4^, ^5^, ^6^, ^7^ 5‐HT neurons also release neurotransmitters from extrasynaptic sites, including the soma. This release mode is known for other monoamine neurotransmitters as well and is termed volume transmission.^8^, ^9^

Administration of glucocorticoids triggers a rapid increase in extracellular 5‐HT concentration in vivo, which is blocked by the glucocorticoid receptor (GR) antagonist mifepristone.^10^ This 5‐HT release occurs too quickly to be mediated by the slow, genomic pathway of glucocorticoid action,^11^ and is more likely caused by rapid, non‐genomic GR actions on vesicle release.^12^ Glucocorticoids were shown to have various rapid effects on 5‐HT neurons, for example, impacting 5‐HT synthesis by affecting tryptophan hydroxylase 2 mRNA levels or causing a rapid upregulation of the cell surface expression of the 5‐HT transporter.^13^, ^14^ However, it remains unknown how glucocorticoids rapidly trigger 5‐HT release.

We employed murine stem cell‐derived 5‐HT neurons^15^, ^16^ to demonstrate that GR resides in close proximity to 5‐HT and vesicle release sites, which is a prerequisite for rapid action of GR on vesicular 5‐HT liberation. We then show that GR activation initiates rapid vesicular 5‐HT release which depends on calcium influx via L‐type VGCC. Our findings provide a more detailed picture of the rapid, glucocorticoid‐induced vesicle release in 5‐HT neurons.

## 2 METHODS

### 2.1 Cell culture

1C11 cells were cultured according to standard protocol.^15^, ^16^ Briefly, 1C11 cells were kept on 100 mm plates (Sarstedt) in DMEM Glutamax with 10% fetal bovine serum, 1% non‐essential amino acids, 1% penicillin/streptomycin, and 1% L‐glutamine (all Life Technologies) at 37°C and 5% CO_2_. For differentiation to serotonergic 1C11 cells (1C11^5‐HT^), 40,000 cells were transferred to a 3.5 cm plate (Sarstedt) containing 3 coverslips or slide chambers (Ibidi) for live cell imaging. Then, culture medium was supplemented with 1 mM dibutyryl cyclic adenosine monophosphate (cAMP) and 0.05% cyclohexanecarboxylic acid for 4 days. On day 4, 1C11^5‐HT^ were shown to display a complete 5‐HT metabolism.^16^ Fetal bovine serum in the medium, which contains 5‐HT, does not impact differentiation to a complete 5‐HT phenotype, but reduces 5‐HT synthesis, content and uptake by activation of 5‐HT_2B_ and 5‐HT_1B/D_ autoreceptors.^16^ As we did not quantify 5‐HT synthesis, content or uptake, this did not impact on our experiments. Additionally, medium was removed, and cells were washed three times in 1x phosphate‐buffered saline (PBS) prior to all experiments to remove medium residues and avoid any bias caused by 5‐HT in the medium. For each treatment, a randomized three‐digit code was applied before image acquisition. Image acquisition and analysis were performed under blinded conditions.

### 2.2 Colocalization analysis

For immunostainings, 1C11^5‐HT^ were fixed in 1% paraformaldehyde and subsequently permeabilized using 0.1% saponin in blocking solution (10% horse serum; 0.2% gelatin in 1x PBS). Antibodies were incubated in blocking solution supplemented with 0.01% saponin for 60 min at room temperature (all chemicals from Sigma Aldrich). Primary antibodies were: GR (mouse; Santa Cruz (sc‐1004); 1:200; specificity assessed by Weber et al.^17^); synaptotagmin 1 (guinea pig; Synaptic Systems (105 105); 1:250; specificity assessed by Shinoda et al.^18^); 5‐HT (rabbit; Sigma Aldrich (S5545); 1:200; specificity assessed by Pelosi et al.^19^). Then, cells were washed with blocking solution, incubated with secondary, Alexa Fluor‐conjugated antibodies (all Thermo Fisher Scientific; donkey‐anti‐rabbit Alexa568 (A10042); goat‐anti‐guinea pig Alexa488 (A‐11073); donkey‐anti‐mouse Alexa488 (A‐21202)) and mounted using Dako Fluorescence Mounting Medium (Dako Cytomation). Images were acquired using a Leica TCS SP5 Confocal imaging system. Confocal z‐stacks were imported into ImageJ, and z‐projections were created for image presentation, determination of region of interest for fluorescence measurements and quantification of vesicular structures.^20^ Colocalization experiments were independently conducted at least three times each on different days using different batches of 1C11. For each experimental condition, images were derived from multiple coverslips or wells, and each cell was captured once. Colocalization was quantified for fluorescent signals on neurites, as high signal densities on somata impeded pixel‐based colocalization analysis, using the colocalization plugin, which calculates Pearson's Correlation Coefficient (PCC). PCC measures the degree to which signal intensities in two channels are linearly correlated to each other. Thus, it is a measure of co‐occurrence and proportionality of signal intensities. PCC of 1 depicts a perfect positive correlation, 0 depicts no correlation, and −1 depicts a perfect negative correlation. It is not impacted by image background and was chosen to quantify colocalization of GR, synaptotagmin 1, 5‐HT and FM4‐64FX because of its robustness and use in previous works.^21^ As proposed by McDonald and Dunn,^22^ one‐sample, one‐tailed Student's *t*‐test was used to determine if PCC was significantly greater than 0 (no correlation).

### 2.3 Fluorescence microscopy

Fluorescent images were acquired using a Leica TCS SP5 Confocal imaging system on a DM IRE2 microscope, equipped with an acusto‐optical beam splitter, an argon ion laser (458–514 nm), a diode‐pumped solid‐state laser (561 nm), and a helium neon laser (633 nm). Laser lines were used as recommended for the applied dyes. For FM4‐64FX staining, 1C11^5‐HT^ grown on coverslips were kept in imaging buffer (130 mM NaCl; 20 mM HEPES; 10 mM glucose; in ddH2O; in dependency of the culture medium, buffer pH was adjusted to 7.2–7.4 with 3 M NaOH solution; all chemicals from Sigma Aldrich) before and after application of either imaging buffer supplemented with 2 µM FM4‐64FX (Thermo Fisher Scientific (F34653); control condition) or reaction buffer (treatment condition). Reaction buffer contained either high potassium or dexamethasone in absence or presence of calcium ions (60 mM KCl or 20 nM dexamethasone; 5 or 0 mM CaCl_2_; 130 mM NaCl; 20 mM HEPES; 10 mM glucose; in ddH2O; in dependency of the culture medium, buffer pH was adjusted to 7.2–7.4 with 3 M NaOH solution; all chemicals from Sigma Aldrich) and was also supplemented with 2 µM FM4‐64FX. Cells were kept in the imaging buffer or reaction buffer supplemented with 2 µM FM4‐64FX for up to 5 min at 37°C. After washing out the fluorescence dye with imaging buffer, cells were fixed with 1% paraformaldehyde and mounted using Dako Fluorescence Mounting Medium (Dako Cytomation) after counterstaining for synaptotagmin 1 or GR (see Section 2.2). Data S1 provides a schematic set‐up of the experiment.

For FFN511 live cell imaging, 1C11^5‐HT^ grown in Ibidi imaging chambers were kept in imaging buffer at 37°C, which was supplemented with 10 µM FFN511 (Abcam (ab120331)) for 10 min. Release of FFN511 was induced by exchanging imaging with reaction buffer for 5 min at 37°C. For buffer exchange, imaging chambers were kept on the heated microscope stage (Ibidi). The GR antagonist mifepristone (10 µM) or VGCC inhibitors in the following concentrations were applied for 30 min prior to loading with FFN511 in presence of the respective inhibitor: GVIA (N‐type; 10 µM); MVIIC (P/Q‐type; 10 µM); nifedipine (L‐type; 10 µM); SNX482 (R‐type; 10 µM). Each cell was exposed to one VGCC inhibitor only. For calcium imaging, cells were maintained as for FFN511 live cell imaging. Imaging buffer was supplemented with 2 µM Fluo4‐AM (Thermo Fisher Scientific (F14201)).^23^ After 30 min, Fluo4‐AM‐containing buffer was exchanged with dye‐free imaging buffer for another 30 min. In case of application of VGCC inhibitors, inhibitors were already added during this incubation time. Then, imaging chambers were perfused with reaction buffer containing either high potassium or dexamethasone in absence or presence of VGCC inhibitors for 5 min.^20^ Images were acquired after FFN511 or Fluo4‐AM loading (control condition) and after application of reaction buffer of interest (treatment condition) as single confocal sections with a layer thickness of 0.49 µm and a high scanning frequency of 800 Hz. The exchange of buffers in the live cell imaging chambers sometimes caused slight shifts in cells’ positions. Thus, it was ensured that the neuron captured in the control condition was still in the image field during treatment condition. A priori sample size calculation was based on an assumed reduction in FFN511 intensity from 100% (control) to 40% (treatment) and a standard deviation of 15% based on previous works for stimulated 1C11^5‐HT^.^20^ A Bonferroni correction for multiple testing with five comparisons (six groups) was applied based on an one‐sided type 1 error of alpha =0.05 (resulting in an alpha/5 = 0.01). For a power of 96% to detect the difference between two groups (assuming non‐parametric tests), a total of *n* = 24 with at least *n* = 4 in each group was required (G*Power V3.1). Image analysis was performed using ImageJ as described above. All experiments were independently conducted at least three times on different days using different batches of 1C11. For each experimental condition, multiple coverslips or wells were used, and each cell was captured once only. All data points were included in the analysis and no outliers were defined. To generate charts of normalized data and perform statistical analysis with raw data, data files were imported into GraphPad Prism7 (GraphPad Software, Inc.). Data were assessed for normality using a normal QQ plot and the Shapiro‐Wilk test. For comparisons of quantifications of FM4‐64FX, FFN511 and Fluo4‐AM signals, Kruskal‐Wallis test and Dunn's multiple comparisons test was performed using the raw data acquired during the experiments. The significance cut‐off (*p*) was 0.05. Data were expressed as median with limits of the interquartile range (25th and 75th percentile).

## 3 RESULTS

### 3.1 GR colocalizes with 5‐HT and synaptotagmin 1

**FIGURE 1:**
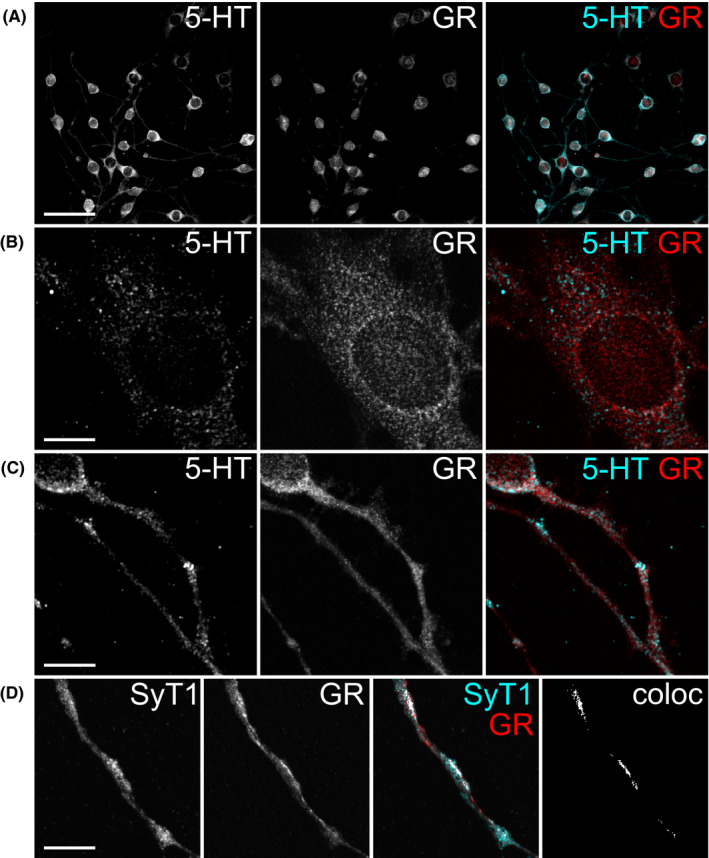
Colocalization of GR with 5‐HT and synaptotagmin 1 in 1C11^5‐HT^. Cellular distribution of GR, 5‐HT and synaptotagmin 1 (SyT1) was analyzed by double‐immunofluorescence labelling. Cyan and red merges to white color. (A) Overview of 1C11^5‐HT^ double‐stained with GR and 5‐HT antibodies. Scale bar: 100 µm. (B) Perinuclear localization of 5‐HT and cytoplasmatic as well as nuclear localization of GR. Scale bar: 10 µm. (C) Distribution of 5‐HT and GR signals on neurites of 1C11^5‐HT^. Scale bar: 20 µm. These localization patterns indicate close proximity of GR to 5‐HT‐containing structures on somata and neurites of 1C11^5‐HT^, which was confirmed in quantitative colocalization analysis. (D) Colocalization analysis of the vesicle marker SyT1 and GR shows that fluorescence signals of both antibodies colocalize along neurites of 1C11^5‐HT^. In the color merge, colocalizing pixels (coloc) are displayed in white. The binary image shows colocalizing pixels only. Scale bars: 10 µm. All images are *z*‐projections of confocal image stacks

To investigate a possible contribution of GR to 5‐HT release, we first analyzed the localization of GR in comparison to 5‐HT itself and to synaptotagmin 1, a protein involved in vesicular 5‐HT release, with immunofluorescence analysis performed in 1C11^5‐HT^ (Figure 1). In cells double‐stained for GR and 5‐HT, we found that GR localized to cell bodies, including the nucleus, but also to neurites (Figure 1A–C). On the cell bodies and neurites, GR antibody signals consisted of circular structures. Compared to the localization pattern of GR, 5‐HT immunostaining displayed a more compact globular structure on neurites and somata of 1C11^5‐HT^, as it is expected for vesicular localization.^3^, ^24^ Regarding the extranuclear distribution of GR, both antibody signals (GR and 5‐ HT) overlapped and were found in similar structures on neurites and cell bodies. This observation indicates that GR may reside in close proximity to 5‐HT‐containing structures. To quantify the colocalization of GR and 5‐HT fluorescence signals, we determined PCC of fluorescent pixels in both channels for neurites recorded independently (Data S2), which was 0.56 ±.02 (mean ± standard error of the mean (SEM); *n* = 77; when compared to PCC of 0: *t*(76) = 36.41, *p* < 0.001). We further analyzed colocalization of GR with synaptotagmin 1 using double‐immunofluorescence labelling (Figure 1D). Synaptotagmin 1 is located in the membrane of synaptic vesicles and is pivotal for calcium‐dependent vesicle fusion and neurotransmitter release.^25^ Indeed, GR immunofluorescence signals were found to colocalize with globular synaptotagmin 1 signals on cell bodies and neurites. Colocalization analysis of GR and synaptotagmin 1 on neurites of 1C11^5‐HT^ revealed a PCC of 0.73 ± 0.01 (mean ± SEM; *n* = 67; when compared to PCC of 0: *t*(66) = 77.95, *p* < 0.001). Within the limits of conventional confocal microscopy's spatial resolution, our analysis shows that GR immunofluorescence signals localized to cellular structures that also harbor 5‐HT and synaptotagmin 1 immunofluorescence signals, indicating that GR may be localized in such close proximity to 5‐HT release sites to be directly involved in the vesicular release process.

### 3.2 Depolarization with potassium chloride triggers L‐type voltage‐gated calcium channel‐dependent vesicular 5‐HT release

**FIGURE 2:**
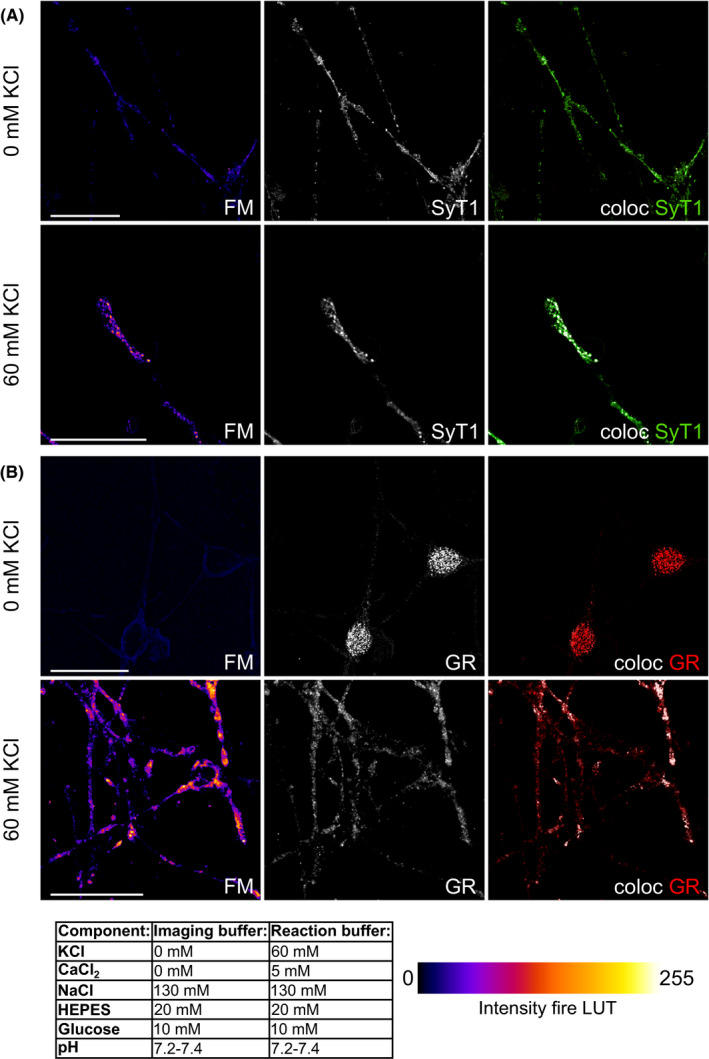
Depolarization via potassium chloride triggers FM4‐64FX dye uptake in synaptic vesicles in 1C11^5‐HT^. 1C11^5‐HT^ were maintained in imaging buffer before application of either imaging buffer supplemented with 2 µM FM4‐64FX (FM; top row in A and B) or reaction buffer, which contained potassium chloride (KCl) and was also supplemented with FM (bottom row in A and B). Buffer components are displayed in the table. After washing out FM dye, cells were fixed in paraformaldehyde and counterstained for either synaptotagmin 1 (SyT1) or GR. (A) KCl‐triggered FM uptake by 1C11^5‐HT^. Counterstaining for SyT1 revealed overlap of both fluorescence signals, indicating that application of high KCl induces vesicular release and uptake of FM dye into vesicles undergoing exocytosis and endocytosis. The colocalization image displays SyT1 signals in green, and pixels where SyT1 and FM signals overlap as white pixels (coloc), FM signals without overlap with SyT1 signals are not displayed. Scale bars: 25 µm. (B) Just like in the top panel, KCl‐triggered uptake of FM. Counterstaining for GR showed overlap of both fluorescent signals, which was confirmed in quantitative colocalization analysis. This indicates spatial proximity of the GR and vesicles undergoing exocytosis and endocytosis. In the colocalization image, GR signals are displayed in red, and pixels where FM and GR signals overlap are shown as white pixels (coloc), FM signals without overlap with GR signals are not displayed. Scale bars: 25 µm

Up to date, the molecular framework how GR activation causes a rapid release of 5‐HT on the cellular level is not completely understood. To provide more insight into this process, we employed 5‐HT neuron‐mimicking 1C11^5‐HT^. As reported previously, high potassium‐induced vesicular 5‐HT release from 1C11^5‐HT^ can be visualized directly either by release of fluorescent false neurotransmitters (FFN)^26^ or by uptake of FM dyes (eg, FM4‐64FX) into vesicles undergoing neurotransmitter release.^3^, ^27^ Figure 2 shows exemplary confocal images acquired for visualization of vesicular 5‐HT release by 1C11^5‐HT^. After exposure to high potassium concentrations, FM4‐64FX was found to localize in globular structures on the cell soma as well as on neurites. Subsequent immunostaining for the vesicle‐associated synaptotagmin 1 revealed that both fluorescence signals overlapped (Figure 2A), verifying that FM4‐64FX was taken up by vesicles releasing 5‐HT. After exposure to high potassium concentrations and counterstaining for GR (Figure 2B), colocalization analysis revealed a PCC of 0.57 ± 0.01 (mean ± SEM; *n* = 222; when compared to PCC of 0: *t*(221) = 60.46, *p* < 0.001), emphasizing that the GR resides in proximity to neurotransmitter release sites.

**FIGURE 3:**
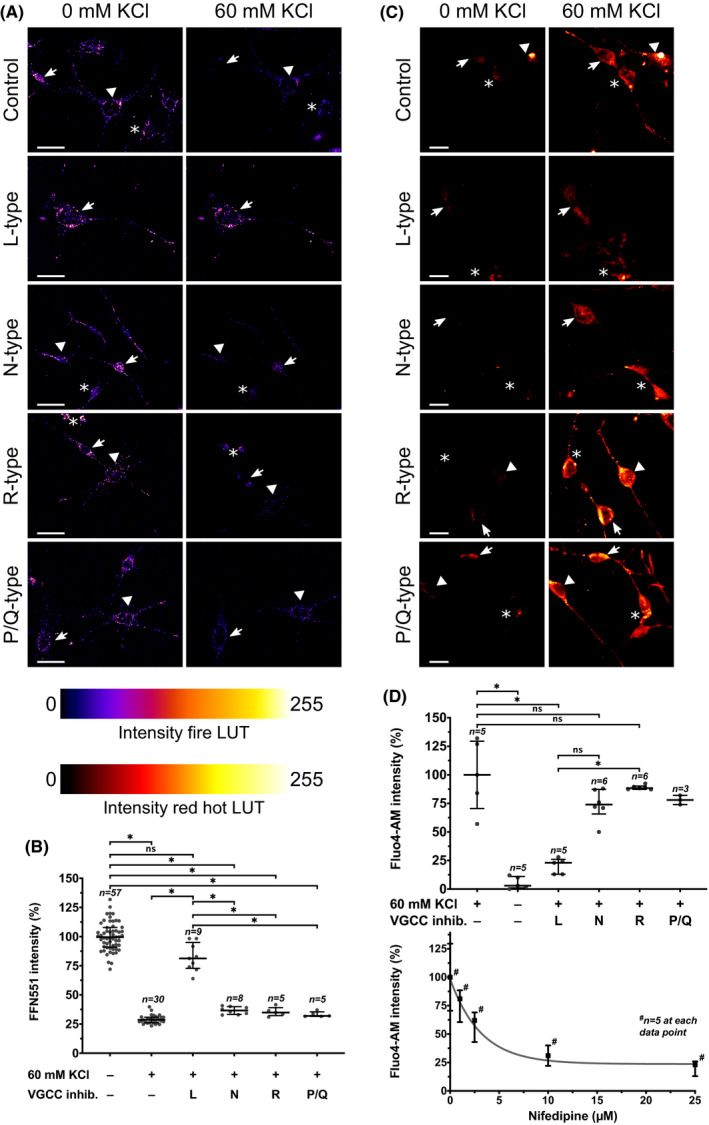
Calcium influx via L‐type voltage‐gated calcium channels after depolarization triggers vesicle release in 1C11^5‐HT^. (A) FFN511 staining of 1C11^5‐HT^ showed a similar distribution pattern as observed for synaptotagmin 1. Cells treated with either N‐type, R‐type or P/Q‐type voltage‐gated calcium channel (VGCC) inhibitors (inhib.) showed similar FFN511 release upon depolarization compared to cells unexposed to VGCC inhibitors (each cell was exposed to one VGCC blocker only). Inhibition of L‐type VGCCs, however, prevented potassium chloride (KCl)‐provoked FFN511 release. Extracellular calcium concentration was 5 mM. Within panels of each VGCC inhibitor, arrows and asterisk depict the same neurons; for the P/Q‐type VGCC inhibitor, the top neuron dislocated during buffer exchange. Scale bars: 25 µm. (B) Kruskal‐Wallis test revealed significant differences between groups (*χ* ^2^(5, *N* = 114) =90.88, *p* < 0.001). Post‐hoc analysis using Dunn's multiple comparison test showed no significant difference in FFN511 signal intensity between resting cells and L‐type VGCC inhibitor‐exposed, stimulated cells (*p* = 0.752), whereas stimulated cells exposed to other VGCC blockers significantly lost FFN511 signal intensity (0 mM KCl vs. 60 mM KCl and N‐type VGCC blocker (*p* = 0.006); 0 mM KCl vs. 60 mM KCl and R‐type VGCC blocker (*p* = 0.03); 0 mM KCl vs. 60 mM KCl and P/Q‐type VGCC blocker (*p* = 0.011)). Results of 3 independent live cell imaging experiments are shown, in which a total of 114 cells were analyzed (*n*_1_ = 35; *n*_2_ = 40; *n*_3_ = 39). Data are displayed as median and interquartile range. (C) Fluo4‐AM was employed to visualize high KCl‐triggered calcium influx. While treatment with neither N‐type, R‐type or P/Q‐type VGCC inhibitors significantly diminished calcium influx upon depolarization, treatment with L‐type VGCC inhibitors strongly reduced calcium ion influx (each cell was treated with one VGCC blocker only). Extracellular calcium concentration was 5 mM. Scale bars: 25 µm. (D) Kruskal–Wallis test revealed significant differences between groups (*χ* ^2^(4, *N* = 27) =21.57, *p* < 0.001). Post‐hoc analysis using Dunn's multiple comparison test showed no significant difference in Fluo4‐AM intensity between stimulated 1C11^5‐HT^ unexposed to VGCC inhibitors and stimulated 1C11^5‐HT^ exposed to either N‐type (*p* = 0.788) or R‐type (*p* > 0.999) VGCC inhibitors (for the P/Q‐type VGCC inhibitor, only descriptive data is presented). Treatment with a L‐type VGCC inhibitor significantly diminished calcium influx upon stimulation (*p* = 0.034 compared to 60 mM KCl without VGCC inhibitor). Results of 3 independent live cell imaging experiments are shown, in which a total of 30 cells were analyzed (*n*_1_ = 8; *n*_2_ = 8; *n*_3_ = 14). Data are displayed as median and interquartile range. The dose‐response curve for nifedipine revealed a dose‐dependent reduction of L‐type VGCC‐mediated calcium influx. 25 cells were analyzed, 5 for each concentration. Data are displayed as median and interquartile range. Descend in Fluo4‐AM intensity was modelled using non‐linear regression (one phase exponential decay; *K* = 0.32). Significance levels: ns (*p* > 0.05); ^*^(*p* ≤ 0.05)

As shown for FM4‐64FX stainings, FFN511‐containing vesicles were located on neurites and cell bodies of 1C11^5‐HT^. After exposure to high potassium concentrations, FFN511 fluorescence intensities significantly dropped due to vesicular dye release (example images in Figure 3A and data analysis Figure 3B; Kruskal–Wallis test (*χ* ^2^(5, *N* = 114) = 90.88, *p* < 0.001; post‐hoc Dunn's multiple comparison test: *p* < 0.001). As expected, high potassium concentrations also induced calcium influx required for neurotransmitter release, which was visualized using Fluo4‐AM (example images in Figure 3C and data analysis in Figure 3D; Kruskal‐Wallis test (χ^2^(4, *N* = 27) = 21.57, *p* < 0.001; post‐hoc Dunn's multiple comparison test: *p* = 0.001). In order to verify that FFN511 release was calcium‐dependent, we employed selective inhibitors for VGCCs and quantified their effect on FFN511 release. As a prerequisite for vesicular release, we also visualized the required calcium influx using Fluo4‐AM. Compared to control conditions, selective inhibitors for the N‐, P/Q‐ and R‐type VGCCs did not diminish potassium chloride‐induced FFN511 release from 1C11^5‐HT^ (exemplary images in Figure 3A and data analysis in Figure 3B; post‐hoc Dunn's multiple comparison test: 0 mM potassium chloride versus 60 mM potassium chloride plus N‐type (*p* = 0.006), R‐type (*p* = 0.03), or P/Q‐type (*p* = 0.011) VGCC inhibitor). In line with the observed effect on FFN511 release, calcium influx was not significantly diminished when N‐type or R‐type VGCC inhibitors were applied (exemplary images in Figure 3C and data analysis in Figure 3D; post‐hoc Dunn's multiple comparison test: 60 mM potassium chloride versus 60 mM potassium chloride plus N‐type (*p* =.788) or R‐type (*p* >.999) VGCC inhibitor; for the P/Q‐type VGCC inhibitor, only descriptive data are presented). In contrast to the latter findings, inhibition of L‐type VGCCs with nifedipine resulted in a significant reduction of FFN511 release from 1C11^5‐HT^ (Figure 3A and B; post‐hoc Dunn's multiple comparison test: 60 mM potassium chloride plus L‐type VGCC inhibitor versus 0 mM potassium chloride (*p* =.752) and versus 60 mM potassium chloride without VGCC (*p* =.006). Furthermore, a dose‐dependent reduction of Fluo4‐AM fluorescence intensity indicated a reduced calcium ion influx when trying to evoke 5‐HT release in the presence of an L‐type VGCC inhibitor (Figure 3D; post‐hoc Dunn's multiple comparison test: 60 mM potassium chloride vs. 60 mM potassium chloride plus L‐type VGCC (*p* =.034)). Taken together, these observations show that L‐type VGCCs mediated calcium ion influx upon depolarization with potassium chloride required to elicit vesicular 5‐HT release in vitro.

### 3.3 GR activation triggers L‐type voltage‐gated calcium channel‐dependent vesicular 5‐HT release

**FIGURE 4:**
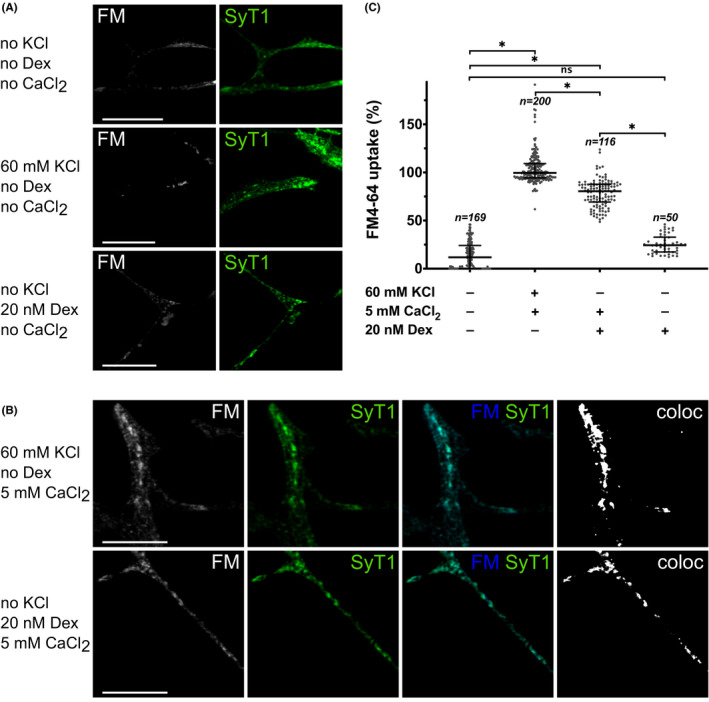
GR activation causes vesicular exocytosis only in the presence of extracellular calcium. (A) Exemplary images for high potassium chloride (KCl)‐induced and dexamethasone (Dex)‐induced FM4‐64FX (FM) uptake in the absence of extracellular calcium (CaCl_2_). Counterstaining with synaptotagmin 1 (SyT1) visualized vesicular structures. Without calcium, neither KCl nor Dex‐induced significant vesicular uptake of FM dye. Scale bars: 5 µm. (B) Exemplary images for high KCl‐induced and Dex‐induced FM dye uptake in the presence of extracellular CaCl_2_. Counterstaining with SyT1 revealed overlapping signals with FM, confirming vesicular uptake of FM. Colocalizing pixels are shown in the binary colocalization images (coloc). Scale bars: 5 µm. (C) Quantification of FM uptake in 1C11^5‐HT^. Kruskal‐Wallis test showed significant differences between groups (*χ* ^2^(3, *N* = 535) =449, *p* < 0.001). Post‐hoc analysis using Dunn's multiple comparison test revealed that in the presence of CaCl_2_, high KCl as well as Dex‐induced significant dye uptake (both *p* < 0.001). In the absence of CaCl_2_, Dex‐induced no significant dye uptake (control vs. 20 nM Dex without CaCl_2_ (*p* = 0.148)). Results of 3 independent experiments are shown, in which a total of 535 cells were analyzed (*n*_1_ = 200; *n*_2_ = 180; *n*_3_ = 155). Data are displayed as median and interquartile range. Significance levels: ns (*p* > 0.05); ^*^(*p* ≤ 0.05)

**FIGURE 5:**
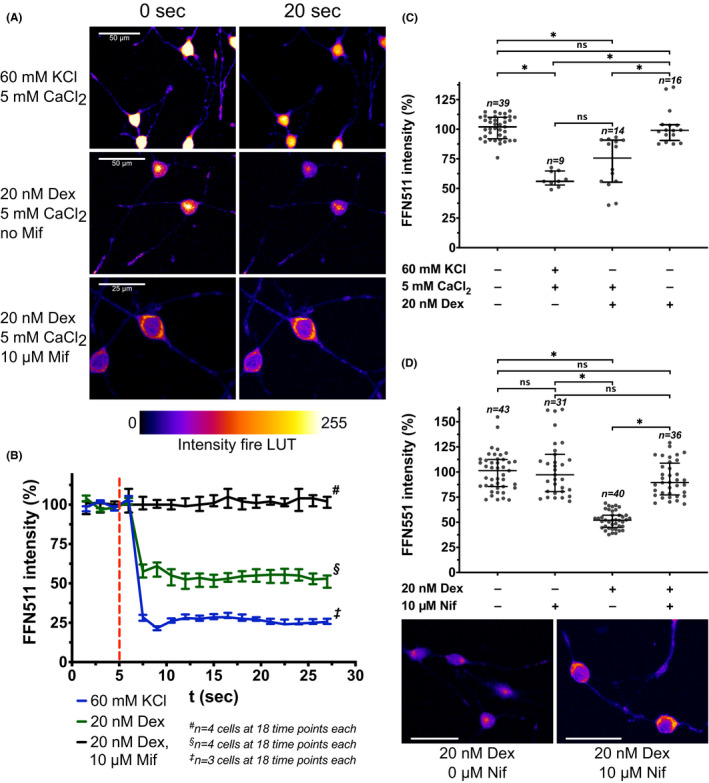
GR activation results in exocytosis mediated by calcium influx via L‐type VGCCs in 1C11^5‐HT^. (A) Exemplary live cell imaging recordings of FFN511 fluorescent signals in 1C11^5‐HT^ before (0 s) and after (20 s) the application of either high potassium chloride (KCl)− or dexamethasone (Dex)‐containing reaction buffer with calcium (CaCl_2_). Incubation with 10 µM mifepristone (Mif) for 30 min inhibited Dex‐induced FFN511 release. Scale bars: as indicated, either 50 µm or 25 µm. (B) Exemplary live cell imaging experiment, where FFN511 intensity was measured every 1.5 s for a total of 27 s (18 measurements per cell in 11 cells total). Each cell received one treatment condition only. Dashed line indicates application of the respective reaction buffer. Data are displayed as median and interquartile range. (C) Quantification of FFN511 release from 1C11^5‐HT^. Kruskal–Wallis test revealed significant differences between groups (*χ* ^2^(3, *N* = 78) =40.29, *p* < 0.001). Post‐hoc analysis using Dunn's multiple comparison test showed that compared to resting cells, FFN511 signal intensities were significantly diminished in the presence of Dex and calcium ions (*p* < 0.001), whereas no significant loss of FFN511 intensity was observed when Dex was applied in the absence of calcium ions (*p* > 0.999). FFN511 intensities were significantly higher in cells exposed to Dex without calcium ions than in cells exposed to Dex with calcium ions (*p* = 0.003). Results of 3 independent live cell imaging experiments are shown, in which a total of 78 cells were analyzed (*n*_1_ = 25; *n*_2_ = 25; *n*_3_ = 28). Data are displayed as median and interquartile range. (D) FFN511 intensity was quantified after the application of Dex and the L‐type VGCC inhibitor nifedipine (Nif). Kruskal‐Wallis test showed significant differences between groups (*χ* ^2^(3, *N* = 150) =88.78, *p* < 0.001). Post‐hoc analysis using Dunn's multiple comparison test showed that 20 nM Dex resulted in a significant reduction of FFN511 intensity (*p* < 0.001), whereas the application of 10 µM Nif inhibited Dex‐induced FFN511 release (control vs. 20 nM Dex and 10 µM Nif (*p* > 0.999)). FFN511 intensities were significantly lower in cells treated with 20 nM Dex than in cells treated with both 20 nM Dex and 10 µM Nif (*p* < 0.001). Results of 3 independent live cell imaging experiments are shown, in which a total of 150 cells were analyzed (*n*_1_ = 50; *n*_2_ = 50; *n*_3_ = 50). Data are displayed as median and interquartile range. Displayed images are exemplary for cells exposed to Dex in presence or absence of Nif. Scale bars: 75 µm. Significance levels: ns (*p* > 0.05); ^*^(*p* ≤ 0.05)

To simulate GR activation in vitro, we applied the selective agonist dexamethasone. Vesicular 5‐HT release upon GR activation with dexamethasone was assessed by performing vesicular uptake experiments with FM4‐64FX. Dexamethasone induced a significant vesicular uptake of FM4‐64FX (Kruskal–Wallis test (*χ* ^2^(3, *N* = 535) = 449, *p* < 0.001; post‐hoc Dunn's multiple comparison test compared to control: *p* < 0.001), which was blocked when calcium was removed from the reaction buffer (*p* = 0.148 compared to control; exemplary images with counterstaining of synaptotagmin 1 in Figure 4A and B, data analysis in Figure 4C). When we applied 20 nM dexamethasone in cells loaded with FFN511, a significant decrease of FFN511 fluorescence intensity was observed, indicating a vesicular, GR‐dependent dye release (Figure 5C; Kruskal–Wallis test (*χ* ^2^(3, *N* = 78) = 40.29, *p* < 0.001; post‐hoc Dunn's multiple comparison test compared to resting cells: *p* < 0.001). Moreover, dexamethasone‐induced FFN511 release occurred within seconds after application, likely indicating a rapid, non‐genomic action of GR (exemplary images in Figure 5A, data analysis in Figure 5B). High potassium‐induced FFN511 release was not affected by prior incubation of 1C11^5‐HT^ with the GR antagonist mifepristone, whereas dexamethasone‐induced FFN511 release was blocked by mifepristone (Figure 5B). Our previous experiments showed that calcium ion influx via L‐type VGCCs contributes to 5‐HT release from 1C11^5‐HT^. Indeed, the absence of calcium ions in the reaction buffer blocked dexamethasone‐induced FFN511 release (Figure 5C; post‐hoc Dunn's multiple comparison test: *p* > 0.999 compared to resting cells). In the next step, we incubated 1C11^5‐HT^ with 10 µM nifedipine to selectively inhibit L‐type VGCCs prior to application of dexamethasone. Comparable to our observation for high potassium‐induced FFN511 release, the inhibition of L‐type VGCCs blocked dexamethasone‐induced FFN511 release from 1C11^5‐HT^ (Figure 5D; Kruskal–Wallis test (*χ* ^2^(3, *N* = 150) = 88.78, *p* < 0.001; post‐hoc Dunn's multiple comparison test of 20 nM dexamethasone plus 10 µM nifedipine compared to resting cells: *p* > 0.999). This suggests that calcium influx via L‐type VGCCs contributes to GR‐dependent, vesicular 5‐HT release in 5‐HT neuron‐mimicking 1C11^5‐HT^.

## 4 DISCUSSION

Previous research suggests that glucocorticoids cause an immediate release of 5‐HT,^10^ but so far, the signaling pathways of glucocorticoid‐induced 5‐HT release are not fully understood. Using murine stem cell‐derived 5‐HT neurons, our results indicate that GR localizes to extranuclear structures in close proximity to 5‐HT release sites. This may be a prerequisite for a non‐genomic interaction with proteins involved in vesicular 5‐HT release. Analogous to high potassium‐induced 5‐HT release, our data provide evidence that activation of GR triggers calcium influx via L‐type VGCCs, which evokes vesicular release of 5‐HT from 1C11^5‐HT^.

A strength of the applied in vitro model is that 1C11^5‐HT^ express most properties of 5‐HT neurons, such as a complete 5‐HT metabolism, and thus mimic the neurochemistry of adult rodent 5‐HT raphé neurons.^15^, ^16^, ^28^, ^29^ Therefore, 1C11^5‐HT^ provide the opportunity to conduct experiments on the molecular level of 5‐HT neurotransmission that are difficult to accomplish in vivo. For example, the arborization of 5‐HT neurons from the raphé nuclei to various brain regions including the hippocampus and prefrontal cortex has constituted a barrier for live cell imaging to track release of 5‐HT vesicles in vivo.^30^ By contrast, vesicular release can easily be visualized in a cellular model such as 1C11^5‐HT^. Additionally, our in vitro model creates an isolated, controlled environment of networks of interacting 5‐HT neurons. Thus, we can exclude the possibility that the effects of dexamethasone on 5‐HT neurons are mediated by other, interacting cells. We analyzed individual, monoclonal 1C11^5‐HT^ grown in networks in wells or on coverslips. While we cannot completely exclude the risk of erroneous external factors impacting batches of 1C11 or individual wells, we mitigated this risk by conducting each experiment on at least three days with new batches of 1C11, and by performing each experiment using multiple wells or coverslips for each condition. However, the 1C11^5‐HT^ in vitro model does not resemble the physiological human or rodent brain environment, which imposes the main limitation on this work. Thus, future experimental work should employ in vivo rodent models to confirm our results.

We employed FM4‐64FX as well as FFN511 to visualize GR‐induced vesicular release in 1C11^5‐HT^, as both dyes are well‐established agents to visualize synaptic, exocytotic events.^3^, ^20^, ^25^, ^26^ Dexamethasone resulted in a rapid increase of FM4‐64FX and decrease of FFN551 signal intensity, similar to depolarization with high potassium. This change in signal intensity was abolished by prior application of the GR antagonist mifepristone, showing that GR activation by a corticosteroid is required to induce vesicular 5‐HT release. In line with the postulated serotonergic volume transmission,^8^, ^9^, ^31^ we were able to detect vesicular release not only at neurite terminals but also on the soma and along neurites of 1C11^5‐HT^.

Our results show that calcium influx is at the center of GR‐induced vesicular 5‐HT release, similar to depolarization‐induced 5‐HT release.^32^, ^33^ Synchronous vesicle exocytosis upon depolarization has been extensively investigated.^4^, ^5^, ^6^, ^34^ Comparable to GR activation, depolarization causes opening of VGCCs with subsequent calcium influx in the active zone of vesicle release.^5^ In this area, vesicles are already docked on the plasma membrane, ready to be released.^4^ Key for docking and priming is the interaction of SNARE proteins, which are either bound to the vesicle membrane (synaptobrevin/VAMP) or the plasma membrane (SNAP‐25 and syntaxin).^5^, ^34^ Within milliseconds after stimulation, calcium binds to the vesicle‐bound synaptotagmin 1, which then binds to the SNARE complex and phospholipids in the plasma membrane, followed by opening of a fusion pore and neurotransmitter release.^4^, ^7^ After release, vesicle recycling either follows clathrin‐mediated endocytosis or fusion pore closing and reuse of already formed vesicles.^6^ Our work suggests that vesicle release upon GR activation might follow a similar mechanism by causing VGCC‐dependent calcium influx, neurotransmitter release and endocytosis. In 1C11^5‐HT^, calcium influx via L‐type is primarily responsible for depolarization‐induced and GR‐induced 5‐HT vesicle release. These results are in line with experimental data by Trueta at al.,^3^ who found that vesicle release in leech 5‐HT neurons is mainly driven by L‐type VGCCs. Similarly, glutamate‐dependent vesicular release from dendrites of 5‐HT neurons was also found to depend on L‐type VGCCs.^35^ Interestingly, a study on dopaminergic neurons, which rely on volume transmission just like 5‐HT neurons,^8^, ^9^ showed that blocking L‐type VGCC with nimodipine abolished depolarization‐induced dopamine release.^36^ Further, GR‐induced 5‐HT release shows similarities to melatonin‐mediated 5‐HT liberation. In more detail, excitation induces 5‐HT release, but the release response increases when neurons are simultaneously exposed to melatonin. As this melatonin‐mediated enhancement of 5‐HT release is abolished by incubation with the L‐type VGCC blocker nifedipine, it was hypothesized that the melatonin receptor is coupled with L‐type VGCCs, thereby facilitating 5‐HT release.^37^ As a limitation, however, this study cannot exclude that VGCCs other than the L‐type VGCC contribute to calcium‐induced vesicular release in 1C11^5‐HT^. While inhibition of the L‐type VGCC significantly reduced vesicular release and calcium influx, it did not completely abolish it. Thus, future research needs to compare the contribution of different VGCCs to vesicular 5‐HT release.

The colocalization between GR, 5‐HT, synaptotagmin 1, which resides in the membrane of neurotransmitter vesicles, and the styryl vesicle dye FM4‐64FX suggests that GR might have a relatively direct, non‐genomic effect on the opening of L‐type VGCC in proximity to vesicle release sites, as it is hypothesized for the melatonin receptor.^37^ Considering the extranuclear GR localization, especially on neurites, this subcellular population of GR molecules may not contribute to genomic events at all. A study by Di et al.^38^ suggests that this GR population is bound to the cell membrane, as dexamethasone bound to bovine serum albumin, which cannot cross cell membranes, still caused a rapid release of endocannabinoids. Further evidence for membrane‐bound GR is provided by a study using electron microscopy, which detected the presence of GR in postsynaptic membrane densities.^39^ Alternatively, it has been proposed that rapid glucocorticoid effects are mediated by cytosolic GR, which associates with the cell membrane similar to the intracellular estrogen receptor.^40^ Possible downstream signaling pathways of rapid GR actions are the G protein–cAMP–protein kinase A (PKA) pathway as well as the mitogen‐activated protein kinase (MAPK) pathway.^41^, ^42^ While we demonstrate a spatial proximity between GR and structures involved in vesicular 5‐HT release, this does not allow to deduce an interaction between GR and individual molecules or structures. Such conclusions require high resolution microscopy and proof of protein‐protein interactions. Hence, future research needs to focus on the identification of direct interaction partners of activated GR and on the molecular pathway of GR‐mediated activation of L‐type VGCCs that ultimately causes rapid vesicular 5‐HT release.

## 5 CONCLUSION

This work extends our understanding how glucocorticoids interact with 5‐HT neurons. In murine stem cell‐derived 5‐HT neurons, the proximity of extranuclear GR, 5‐HT and synaptotagmin 1 suggests that GR might be directly involved in rapid 5‐HT release. Glucocorticoid binding to GR initiates a rapid vesicular 5‐HT release, which depends on extracellular calcium influx via L‐type VGCCs. Extending the knowledge of the acute and chronic effects of glucocorticoids on 5‐HT neurons might ultimately contribute to the understanding of the underlying mechanisms of stress‐related psychiatric disorders.

## CONFLICTS OF INTEREST

The authors declare that the research was conducted in the absence of any financial support or relationships that could be construed as a potential conflict of interest.

## AUTHOR CONTRIBUTIONS

TL, NP, TW and DB contributed to the conception of the study. TL, NP, JR and SL conducted the experiments and analyzed the data. TL and NP prepared the manuscript. All authors contributed to critical revision of the manuscript and approved the submitted version.

## Supporting information

Data S1

Data S2

## Data Availability

The data that support the findings of this study are available from the corresponding author upon reasonable request.
