# Supplementary material for: Activation of the glucocorticoid receptor rapidly triggers calcium‐dependent serotonin release in vitro
Source: CNS Neurosci Ther. 2021 Mar 14;27(7):753–64. doi: 10.1111/cns.13634 (PMC8193689; doi:10.1111/cns.13634)
Supplement: Supplementary file 1 — Data S1 [file CNS-27-753-s002.docx]

**
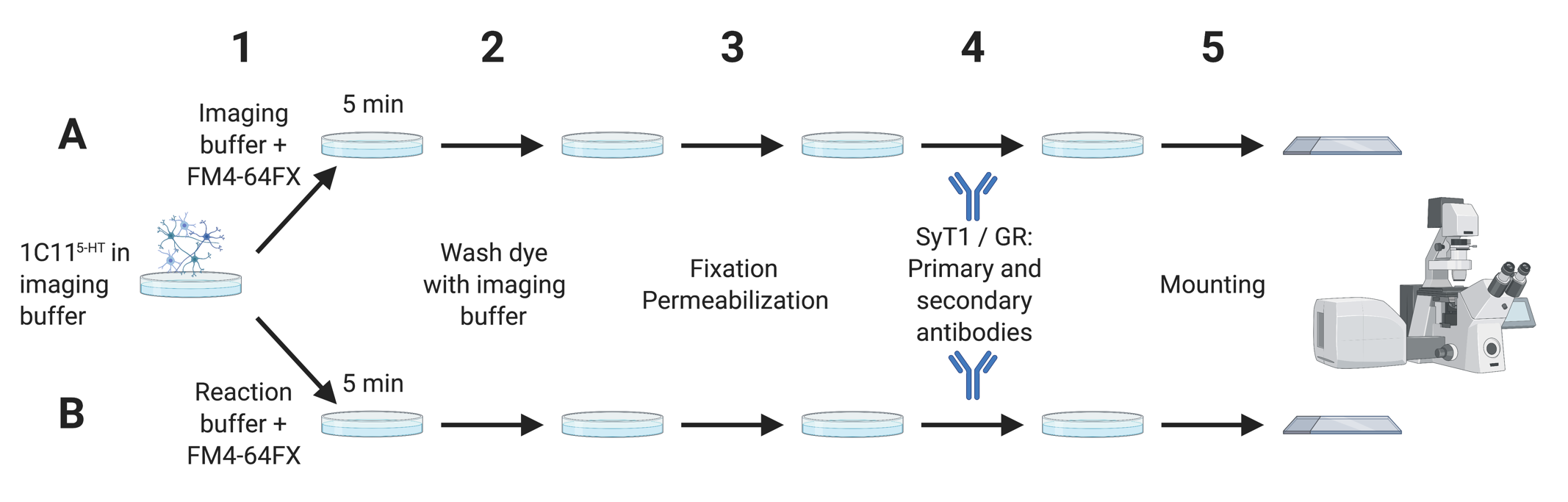
Supplement 1: Schematic experimental set-up for FM4-64FX experiments.** 1C11^5-HT^ were kept in imaging buffer before (1) application of either imaging buffer with 2 µM FM4-64FX (A; control condition) or reaction buffer with 2 µM FM4-64FX containing either potassium chloride or dexamethasone with or without calcium (B; treatment condition) for 5 min. Cells did not receive both, control and treatment condition. (2) After washing out the fluorescence dye with imaging buffer, (3) 1C11^5-HT^ were fixed and permeabilized. (4) Cells were incubated with primary antibodies for either GR or synaptotagmin 1 (SyT1) and secondary, Alexa Fluor-conjugated antibodies. (5) Finally, 1C11^5-HT^ were mounted for fluorescence microscopy.
