# Supplementary material for: Activation of the glucocorticoid receptor rapidly triggers calcium‐dependent serotonin release in vitro
Source: CNS Neurosci Ther. 2021 Mar 14;27(7):753–64. doi: 10.1111/cns.13634 (PMC8193689; doi:10.1111/cns.13634)
Supplement: Supplementary file 2 — Data S2 [file CNS-27-753-s001.docx]

***Supplement 2: Quantification of colocalization of GR and synaptotagmin 1, GR and 5-HT as well as GR and FM4-64FX on neurites of 1C11^5-HT^.*** To determine spatial proximity, colocalization between the fluorescence signals of GR and the vesicular calcium sensor synaptotagmin 1 (SyT1) was quantified on neurites of 1C11^5-HT^. Pearson’s Correlation Coefficient was 0.73 ± .01 (mean ± standard error of the mean (SEM); n = 67). For the colocalization between the fluorescence signals of GR and 5-HT, Pearson’s Correlation Coefficient was 0.56 ± .02 (mean ± SEM; n = 77). Colocalization analysis of the fluorescence signals of GR and the styryl vesicle dye FM4-64FX (FM) revealed a Pearson’s Correlation Coefficient of 0.57 ± .01 (mean ± SEM; n = 222). This indicates spatial proximity between the GR and SyT1, 5-HT and FM, respectively. Graph displays scatter plots with means; error bars depict SEM.
